# Tutorial on methods for estimation of optical absorption and scattering properties of tissue

**DOI:** 10.1117/1.JBO.29.6.060801

**Published:** 2024-06-11

**Authors:** Ran Tao, Janek Gröhl, Lina Hacker, Antonio Pifferi, Darren Roblyer, Sarah E. Bohndiek

**Affiliations:** a University of Cambridge, Department of Physics, Cambridge, United Kingdom; b University of Cambridge, Cancer Research UK Cambridge Institute, Li Ka Shing Centre, Cambridge, United Kingdom; c University of Oxford, Department of Oncology, Oxford, United Kingdom; d Politecnico di Milano, Department of Physics, Milano, Italy; e Boston University, Department of Electrical and Computer Engineering, Boston, Massachusetts, United States; f Boston University, Department of Biomedical Engineering, Boston, Massachusetts, United States

**Keywords:** tissue optics, tissue optical properties, diffuse optics, optics, photonics

## Abstract

**Significance:**

The estimation of tissue optical properties using diffuse optics has found a range of applications in disease detection, therapy monitoring, and general health care. Biomarkers derived from the estimated optical absorption and scattering coefficients can reflect the underlying progression of many biological processes in tissues.

**Aim:**

Complex light–tissue interactions make it challenging to disentangle the absorption and scattering coefficients, so dedicated measurement systems are required. We aim to help readers understand the measurement principles and practical considerations needed when choosing between different estimation methods based on diffuse optics.

**Approach:**

The estimation methods can be categorized as: steady state, time domain, time frequency domain (FD), spatial domain, and spatial FD. The experimental measurements are coupled with models of light–tissue interactions, which enable inverse solutions for the absorption and scattering coefficients from the measured tissue reflectance and/or transmittance.

**Results:**

The estimation of tissue optical properties has been applied to characterize a variety of *ex vivo* and *in vivo* tissues, as well as tissue-mimicking phantoms. Choosing a specific estimation method for a certain application has to trade-off its advantages and limitations.

**Conclusion:**

Optical absorption and scattering property estimation is an increasingly important and accessible approach for medical diagnosis and health monitoring.

## 1 Introduction

Visible (∼400 to 700 nm) and near-infrared-I (NIR-I, 650 to 950 nm) light is widely used to safely and often noninvasively interrogate tissues, as changes in tissue optical properties are frequently associated with the underlying progression of many biological processes in body.^1^^–^^3^ The quantitative measurement of tissue optical properties is challenging due to the complexity of light–tissue interactions. The dominant interactions of light in tissues are optical absorption and scattering events, which determine the measurable transmission and reflection.^1^^–^^4^ Extracting absorption and scattering properties from the measured light transmission and/or reflection, is therefore, an inverse problem requiring computational models of light–tissue interactions and well-designed measurement devices. This tutorial aims to help readers understand the process of extracting optical absorption and scattering properties from a given sample by solving the inverse problem and is structured as follows. Section 2 defines the parameters that are used to quantify tissue optical absorption and scattering properties, Sec. 3 describes the available models of light–tissue interactions, then Secs. 4 and 5 review the typical estimation techniques of tissue optical absorption and scattering properties with their advantages and limitations discussed. The tutorial ends in Sec. 6 with a summary and an outlook to future opportunities.

## 2 Quantifying Tissue Optical Properties

When light is incident on the tissue, it is partially reflected at the tissue/air interface due to the mismatch of refractive index (n). The remaining light then penetrates the tissue and, given a sufficiently large pathlength, experiences multiple scattering and absorption events that spatially broaden and attenuate the light, which may ultimately escape the tissue for detection in transmission or reflection mode. A wide range of other light–tissue interactions may also occur, such as fluorescence, inelastic (Raman) scattering, polarization, and photoacoustic effects, but these are out of the scope of this tutorial. A detailed description of the physics of light–tissue interactions can be found in textbooks,^1^^–^^3^ review papers,^5^ and online lecture notes;^6^ a brief summary, needed to understand the process of estimation of optical absorption and scattering properties, is provided below.

**Fig. 1:**
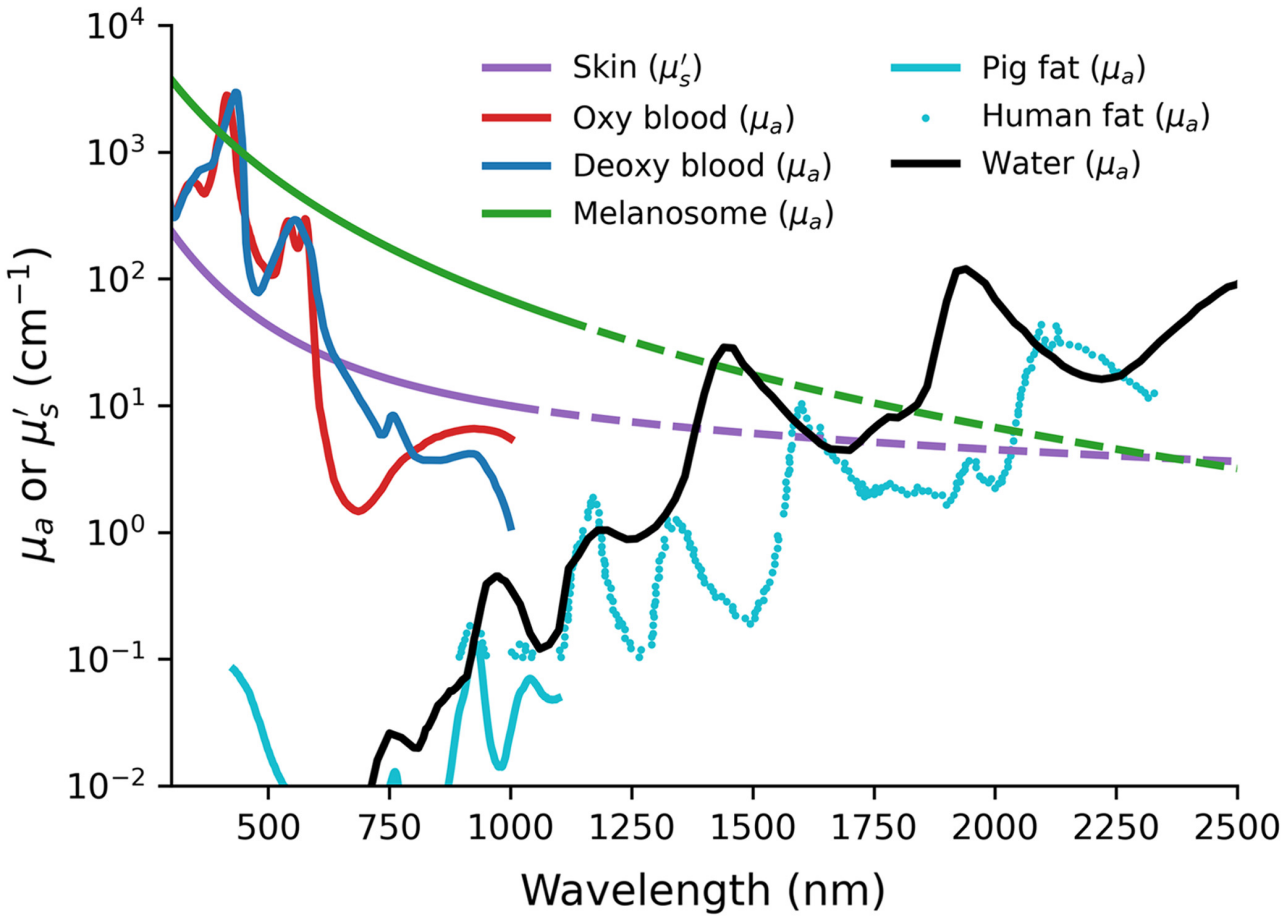
Absorption spectra of the main endogenous tissue chromophores and the skin scattering spectrum. Absorption spectra include: oxygenated (red line) and deoxygenated (blue line) blood with 150 g hemoglobin per liter;^8^^,^^9^ melanosome (green line), of which values beyond 1100 nm are extrapolated from the values at shorter wavelengths;^9^ purified pig fat (cyan line) and filtered human fat (cyan dots);^8^^–^^10^ and pure water (black line).^8^^,^^9^^,^^11^ The skin scattering spectrum (purple line) was modeled by a linear combination of the Mie and Rayleigh expressions, using the parameters in Ref. 9 $\mu_{s}^{\prime }$ values beyond 1000 nm are extrapolated using the modeled expression.

The optical absorption coefficient ($\mu_{a}$) is defined as the probability of photon absorption per unit pathlength, with a typical order of magnitude of $0.1\;{\mathrm{cm}}^{-1}$ in the NIR-I window.^3^^,^^5^ Absorption occurs in tissue chromophores, and $\mu_{a}$ is a linear combination of the molar extinction coefficients for all chromophores present, weighted by their concentrations. The dominant chromophores in blood at visible and NIR-I wavelengths are oxyhemoglobin and deoxyhemoglobin. The ratio of oxyhemoglobin concentration to the total hemoglobin concentration is defined as the oxygen saturation, which is an important biomarker of interest in many clinical applications, such as tissue oxygenation monitoring.^3^^,^^7^ Other chromophores include melanin, water, lipids, and collagen. A set of pure absorption spectra of common tissue chromophores has been collated and is widely used in the field as a reference (Fig. 1).^8^ By knowing the chromophore absorption spectra, $\mu_{a}$ can be decomposed to quantify the concentrations of each chromophore.

Scattering arises as a result of difference in n between the scatterer (e.g., cell nucleus) and its surrounding medium. The probability of photon scattering per unit pathlength defines the optical scattering coefficient ($\mu_{s}$), with a typical order of magnitude of $100\;{\mathrm{cm}}^{-1}$ in the NIR-I window.^1^^,^^3^^,^^5^ Therefore, the mean free path between two scattering events (${\mathrm{mfp}}_{s}$) is the inverse of $\mu_{s}$, i.e., $1/\mu_{s}$. In bulk tissues, scattering occurs multiple times, so the scattering angle can be averaged and its average cosine is defined as the anisotropy factor (g=⟨cos θ⟩). g=0 means isotropic scattering, and g=1 means the light propagates forward. The near-forward scattering in most biological tissues implies that several scattering events have to occur before the light becomes truly diffused, which leads to the definition of the reduced scattering coefficient [$\mu_{s}^{\prime }=\mu_{s}(1-g)$]. The mean free path between effectively isotropic scattering events (${\mathrm{mfp}}_{s}^{\prime }$) is $1/\mu_{s}^{\prime }$, which is 1/(1−g) times longer than ${\mathrm{mfp}}_{s}$, showing that multiple single-scattering events have occurred. As g=0.9 is frequently used to represent average tissues, $\mu_{s}^{\prime }$ has a typical order of magnitude of $10\;{\mathrm{cm}}^{-1}$ in the NIR-I window.^1^^,^^3^^,^^5^ Approximating biological structures as spherical scatterers, an empirical derivation based on Mie theory can be used to model the $\mu_{s}^{\prime }$ spectrum and gives a function proportional to $\lambda^{-b}$, where λ is the optical wavelength and b is a positive constant.^12^^,^^13^ When the size of the scatterers is much smaller than λ, the Mie scattering reduces to the Rayleigh scattering, which is proportional to $\lambda^{-4}$. When both large and small scatterers are present, $\mu_{s}^{\prime }$ spectrum can be fitted by a linear combination of the Mie and Rayleigh expressions (Fig. 1).^5^

Table 1 summarizes parameters used to characterize tissue optical properties, along with their derived properties as used in diffusion theory (see Sec. 3.1). Summaries of optical properties including $\mu_{a}$ and $\mu_{s}^{\prime }$ spectra and n measured on human tissues can be found in Refs. 2, 5, and 14.

**Table 1:** Summary of tissue optical properties. The definitions and notations follow those in textbooks Refs. 1 and 3.

	Parameter	Symbol	Definition	Common unit
Absorption	Absorption coefficient	$\mu_{a}$	Probability of photon absorption per unit pathlength	${\mathrm{cm}}^{-1}$ or ${\mathrm{mm}}^{-1}$
Scattering	Scattering coefficient	$\mu_{s}$	Probability of photon scattering per unit pathlength	${\mathrm{cm}}^{-1}$ or ${\mathrm{mm}}^{-1}$
	Scattering mean free path	${\mathrm{mfp}}_{s}$	$\frac{1}{\mu_{s}}$	μm, mm, or cm
	Anisotropy factor	g	Average of cosine of scattering polar angle by single scattering: ⟨cos θ⟩	—
Diffusive regime	Reduced scattering coefficient	$\mu_{s}^{\prime }$	Probability of equivalent isotropic photon scattering per unit pathlength in diffusive regime: $\mu_{s}^{\prime }=\mu_{s}(1-g)$	${\mathrm{cm}}^{-1}$ or ${\mathrm{mm}}^{-1}$
	Reduced scattering mean free path	${\mathrm{mfp}}_{s}^{\prime }$	$\frac{1}{\mu_{s}^{\prime }}$	μm, mm, or cm
	Transport coefficient	$\mu_{t}^{\prime }$	$\mu_{a}+\mu_{s}^{\prime }$	${\mathrm{cm}}^{-1}$ or ${\mathrm{mm}}^{-1}$
	Reduced albedo	$a^{\prime }$	$\frac{\mu_{s}^{\prime }}{\mu_{t}^{\prime }}=\frac{\mu_{s}^{\prime }}{\mu_{a}+\mu_{s}^{\prime }}$	—
	Diffusion coefficient	D	$\frac{1}{3\mu_{t}^{\prime }}=\frac{1}{3(\mu_{a}+\mu_{s}^{\prime })}$	μm, mm, or cm
	Effective attenuation coefficient	$\mu_{\mathrm{eff}}$	$\sqrt{\frac{\mu_{a}}{D}}=\sqrt{3\mu_{a}(\mu_{a}+\mu_{s}^{\prime })}$	${\mathrm{cm}}^{-1}$ or ${\mathrm{mm}}^{-1}$
Refraction and reflection	Refractive index	n	Ratio of speed of light in vacuum to that in medium	—
	Reflection parameter	A	$\frac{1+R_{\mathrm{eff}}}{1-R_{\mathrm{eff}}}$, and $R_{\mathrm{eff}}$ can be approximated by the empirical relationship $-1.440n^{-2}+0.710n^{-1}+0.668+0.0636n$	—

## 3 Modeling of Light–Tissue Interactions

### 3.1 Computational Models

The most straightforward approach to modeling light–tissue interactions employs the Beer–Lambert law.^15^ It relates the energy of a light beam to the length of the path of the beam through the medium and to $\mu_{a}$ and $\mu_{s}$ of the medium. Due to the simplicity of the approach, it is not capable of accurately modeling multiple scattering events, making it inadequate for determining light propagation in bulk scattering media.^1^

A common approach to accurately simulating the propagation of light in tissue is to use the radiative transfer equation (RTE),^16^ which models the propagation of radiation through a medium affected by absorption, emission, and scattering processes.^17^ Several solutions for the RTE have been proposed to date. The most commonly used approximate solution to the RTE is the diffusion theory or diffusion approximation, which is a first-order angular approximation for the RTE. It assumes $\mu_{s}^{\prime }\gg \mu_{a}$ and holds true after a certain number of scattering events, therefore, it fails at very early propagation times (e.g., <100  ps) or in the proximity of collimated light sources.^18^ Many analytical expressions have been derived in different geometries, ranging from the semi-infinite medium, to the slab, from the sphere to the cylinder.^19^

Other methods to solve the RTE include the finite-element method or the adding–doubling (AD) method. Finite-element models are commonly used under the diffusion approximation to tackle inhomogeneous media. Yet, they can be applied also beyond the diffusion approximation employing adaptively refined unstructured grids, making it possible to calculate the radiation field across several length scales.^20^^,^^21^ The AD method can be used to calculate reflectance and transmittance for a sample by iteratively adding and doubling the thickness of sample layers.^22^^,^^23^

Other approaches to modeling photon migration in diffuse media^19^ include random walk,^24^ Feynman path integral,^25^ the telegrapher equation,^26^ higher-order approximation to the RTE beyond the diffusion approximation,^27^ or direct solution to the RTE.^28^

An alternative solution to the RTE is to employ a Monte Carlo (MC) model that stochastically models millions of photon paths through the medium, accounting for photon absorption and scattering.^29^ The MC method is known to describe light propagation accurately, but requires long computation times, limiting its use in iterative optimization schemes. To mitigate the computational restrictions of MC methods, white MC models have been proposed,^30^^–^^32^ in which a single simulation in combination with proper rescaling ensures coverage of a wide range of optical properties. The introduction of scaling relations to quickly regenerate MC simulations for small inhomogeneities in optical properties will speed up the use of MC in inverse problems.^32^^,^^33^ In addition, researchers employ GPU-accelerated MC implementations to maximize the computation speeds^34^ or use precomputed lookup tables^14^ to minimize inversion time.

In summary, multiple strategies and implementations exist to model light–tissue interactions. Choosing a method for any given application includes assessing the adequacy of the method assumptions and a trade-off between the method’s accuracy and computational requirements. For example, diffusion approximation is typically chosen over MC methods when it is possible to trade accuracy close to the detector for orders-of-magnitude higher computation speeds.

### 3.2 Tissue-Mimicking Test Objects for Model Validation

Tissue-mimicking test objects are commonly used tools to validate and optimize models and techniques for the estimation of tissue optical properties.^35^ These test objects are referred to as “phantoms” and provide a controlled, carefully defined experimental (physical or computational) environment that replicates the optical characteristics of specific biological tissues and/or pathological conditions. Thereby, phantoms enable accurate and reproducible measurement outcomes, making them invaluable for testing and validation purposes.

Phantoms are usually tailored toward specific applications and system types so can, therefore, differ widely in shape and complexity. Commonly used phantom base materials for biophotonic applications include: hydrogels,^36^^,^^37^ polyvinyl alcohol (PVA),^38^^,^^39^ polyvinyl chloride plastisol (PVCP),^40^^–^^46^ silicone,^47^^,^^48^ resin-based materials,^49^^,^^50^ and copolymer-in-oil materials.^51^^–^^57^ Each of these material types is characterized by distinct advantages and disadvantages.^35^^,^^58^ Hydrogels have tissue-mimicking properties and a simple manufacturing procedure but are susceptible to mechanical damage, dehydration, and bacterial growth in storage, limiting their shelf life.^59^^–^^61^ PVA cryogels exhibit higher structural rigidity and longevity than hydrogels but have a more complex preparation procedure with long freeze-thaw cycles,^58^ impairing reproducibility.^62^ Resin-based materials and silicone feature long-term stability but their mechanical and acoustic properties deviate from soft tissue,^61^^,^^63^^,^^64^ making them suboptimal for hybrid applications, such as photoacoustic imaging. PVCP faces challenges, such as high preparation temperatures for fabrication,^43^^,^^44^^,^^65^^,^^66^ a limited scientific supply chain,^42^ and the potential use of phthalate-based plasticizers, posing risks of reproductive and developmental toxicity.^67^ Copolymer-in-oil materials exhibit tissue-mimicking properties and long-term stability but have a more complex fabrication procedure than hydrogels.^57^ *Ex vivo* animal tissues or bioengineered phantoms have also been employed to pinpoint optical properties, resembling more closely the light–tissue interactions found in human tissues, but suffer from limited reproducibility and tuneablity.^35^

The molecular composition of the phantom base material determines the types of additives that can be used to tune its optical properties. Additives for adjusting optical scattering can be broadly divided into lipids (such as Intralipid), white metal oxide suspensions, polymer microspheres, and (rarely) gold nanoparticles.^58^ For tuning optical absorption, either natural tissue chromophores (such as hemoglobin or melanin) or synthetic absorbers (such as pigment-based inks like India ink or molecular dyes) can be used. Fabrication techniques for phantoms include molding, casting, and three-dimensional (3D) printing, allowing for the creation of simple to widely complex geometries that cater to the diverse needs of optical measurement techniques.

Phantoms can also cover *in silico* frameworks to form the foundation for numerical forward models of the physical light interactions. For example, the simulation and image processing for photonics and acoustics (SIMPA) toolkit can be used to define arbitrary volumetric distributions of $\mu_{a}$ and $\mu_{s}$ and then simulate light fluence and diffuse reflectance with MC models.^68^ Such *in silico* tissue representations can be especially valuable when they are digital equivalents of real-world phantoms^69^ to enable powerful supervised data analysis strategies.

A standardized protocol, MEDPHOT (optical methods for medical diagnosis and monitoring of diseases), for the assessment of $\mu_{a}$ and $\mu_{s}^{\prime }$ in homogeneous media has been established that is based on a unique matrix of 32 solid epoxy resin phantoms spanning a wide range of optical properties.^70^ India ink and Intralipid are often used as reference materials for liquid phantoms due to their extensive characterization in the literature, also in multilaboratory exercises.^71^^,^^72^ Nonetheless, owing to the wide range of optical imaging applications, broadly applicable standards-for-test objects and methods have not yet been introduced and are the subject of extensive review elsewhere.^35^

## 4 Estimating Tissue Optical Properties

**Fig. 2:**
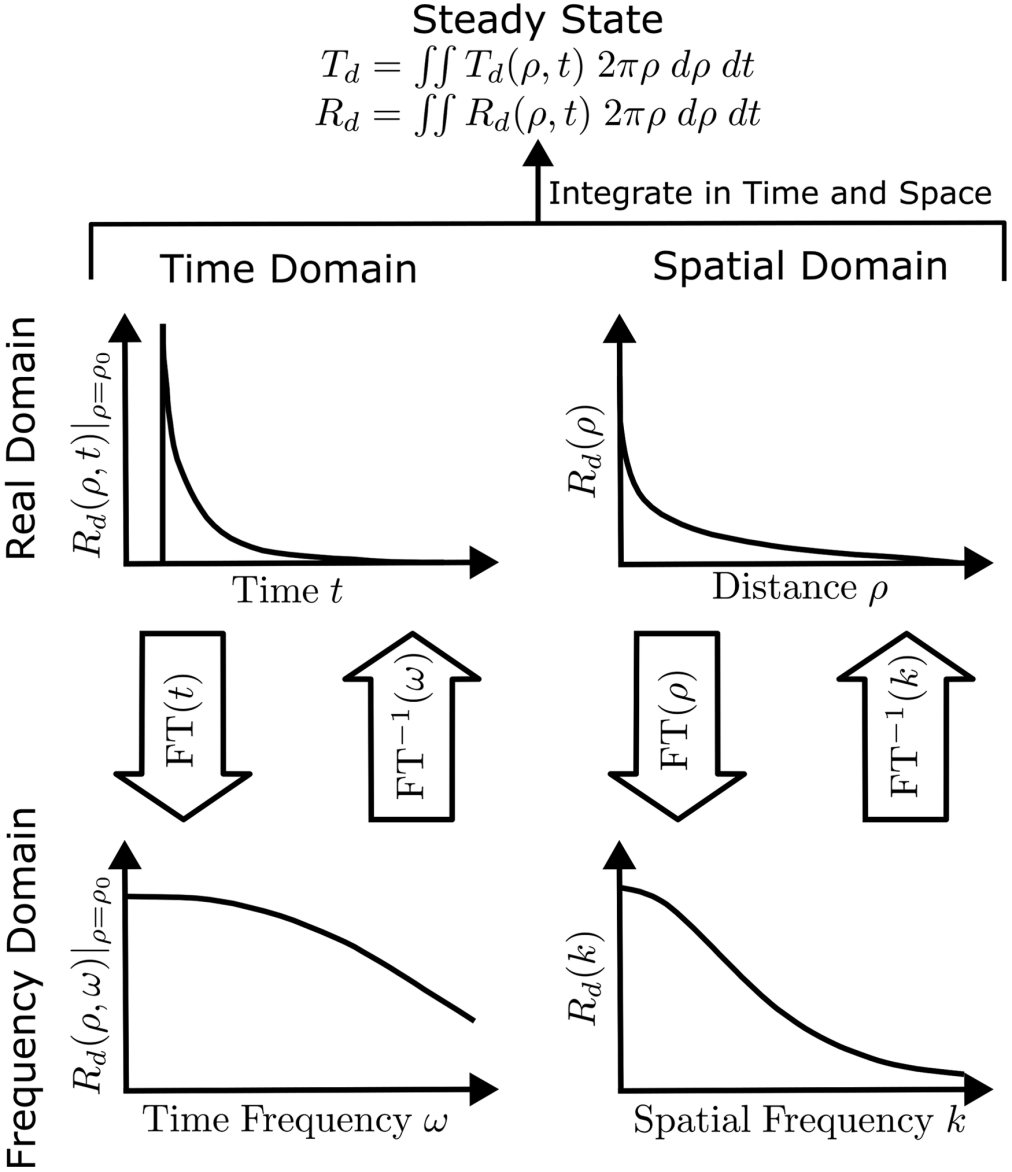
Relationships among different domains [steady state, time domain (TD), time frequency domain (FD), spatial domain, and spatial FD] for tissue optical property estimation. The steady-state measures diffuse transmittance ($T_{d}$) and reflectance ($R_{d}$), which are the integrals of $T_{d}(\rho ,t)$ and $R_{d}(\rho ,t)$ in both the time (t) and spatial (ρ) domains. The real domain and FD are related by FT and inverse Fourier transform (${\mathrm{FT}}^{-1}$). Reproduced with permission from Ref. 73.

Estimations of optical absorption and scattering properties involve measurements that are resolved, collected, analyzed, and interpreted with respect to either time, space, or steady-state domains. Steady-state methods measure the total reflectance and/or transmittance, essentially continuous-wave integrating both in time and space. Measurements conducted in time domain (TD) assess the temporal broadening of a laser pulse propagating through the probed tissue. The temporal response can be equivalently measured in the frequency domain (FD) since the measured frequency response is equivalent to the temporal response through the Fourier transform (FT). Yet, for the full correspondence, a wide range of modulation frequencies must be sampled up to the GHz range. Similarly, the spatial response to a temporally continuous-wave light source can be measured in spatial domain (SD) or spatial FD. Thereby, there are broadly five measurement domains: steady-state domain, TD, time FD, SD, and spatial FD (Fig. 2).

Only estimations of absorption and scattering properties are considered in this tutorial. n and g are usually taken from literature values and typically need separate measurement systems to evaluate their values if unknown. n is different for each microscopic tissue component, and therefore, is the major source of scattering (see Sec. 2). It is the macroscopic average n that is measured for bulk tissue. Measurements of average n are usually based on the fact that discontinuities in n result in refraction and total internal reflection at the critical angle. Refractometers based on the principle of total internal reflection are commonly used to measure n.^2^^,^^4^^,^^5^ Interferometry is another method to measure n by detecting the optical pathlength of a sample with a given thickness.^74^ n depends on the water content, so is in the range from 1.33 ($n_{\text{water}}$) to around 1.5 ($n_{\text{dry}}$).^5^ Soft tissues have similar water contents and hence have a narrow range of n^74^ centering around 1.38,^2^^,^^3^ which is why literature values can typically be taken.

The anisotropy factor g is usually measured by goniometry, where a collimated detector rotates around a thin sample to measure the angular distribution of singly-scattered light and calculates g.^4^^,^^5^ To ensure that only single scattering occurs, the sample should have a thickness less than ${\mathrm{mfp}}_{s}$, typically of 100  μm, which may suffer from desiccation and heterogeneity issues.^5^ The measurements of scattering signal at backward directions are tricky due to the lower probability of backward scattering.^5^ For flat samples, the measurements around ±90 deg are generally missing.^4^ The refraction and reflection of light and other experimental factors can change the direction and intensity of detected signal, which further complicates the measurements.^4^^,^^5^

In terms of measuring optical absorption and scattering properties, the Beer–Lambert law can only be applied to thin samples where only single scattering occurs and is not suitable for bulk scattering tissue measurements (see Sec. 3.1). These thin samples have desiccation and heterogeneity issues as noted above. For bulk tissues, diffuse reflectance ($R_{d}$) and/or diffuse transmittance ($T_{d}$) are measurable quantities, from which $\mu_{a}$ and $\mu_{s}^{\prime }$ are found by inverting a model of light–tissue interactions.

### 4.1 Steady-State Measurement Methods

Steady-state techniques measure the total reflectance and transmittance of a tissue sample under continuous-wave collimated illumination.

#### 4.1.1 Measurement methods

A steady-state reflectance measurement on a semi-infinite medium is not sufficient to decouple $\mu_{a}$ and $\mu_{s}^{\prime }$. From diffusion theory, when the homogeneous semi-infinite medium is illuminated by a collimated point source that covers an infinitesimally small area, such as a laser beam, its $R_{d}$ is^1^

$$R_{d}=\frac{a^{\prime }}{2}e^{-\sqrt{3(1-a^{\prime })}}(1+e^{-\frac{4A}{3}\sqrt{3(1-a^{\prime })}}),$$ (1)

where $a^{\prime }$ and A are defined in Table 1.

Following a different application angle, for the case of plane-wave light normally incident onto the sample surface for broad-beam wide-field illumination, say a collimated light-emitting diode (LED) or halogen light, then $R_{d}$ can be expressed as^1^^,^^4^^,^^75^

$$R_{d}=\frac{a^{\prime }}{1+2A(1-a^{\prime })+(1+\frac{2A}{3})\sqrt{3(1-a^{\prime })}}.$$ (2)

The two expressions—though derived from different assumptions—collapse to the same formula in diffusive regime after approximating Eq. (1) with the lowest orders in its Taylor expansion.^1^ For both cases, only $a^{\prime }$ can be calculated, and $\mu_{a}$ and $\mu_{s}^{\prime }$ cannot be separated. To separate $\mu_{a}$ and $\mu_{s}^{\prime }$, at least two measurements are required, so both $R_{d}$ and $T_{d}$ should be measured, which means that the tissue sample should be of proper thickness so that bulk $T_{d}$ can be measured, and therefore, often only *ex vivo* measurements can be performed in steady state.

**Fig. 3:**
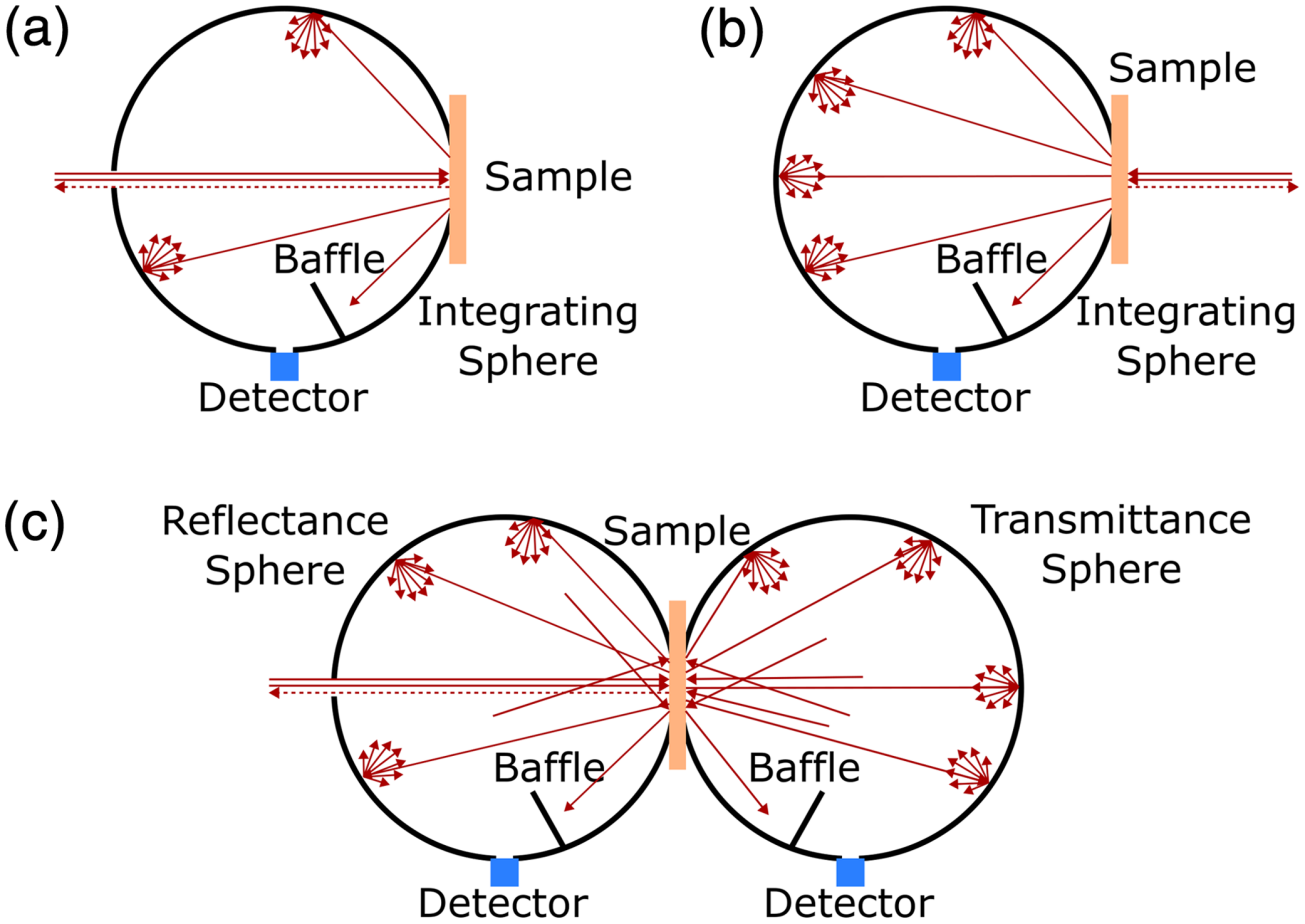
Schematics of methods for steady-state measurements. A single-integrating sphere can be used to measure (a) the reflectance and (b) the transmittance. Alternatively, (c) two spheres can be configured such that the reflectance and transmittance are measured simultaneously. The illumination beam in this figure is at 0 deg, but 8 deg incident angle is frequently used to include the specular reflection. Reflectance and transmittance spectra can be measured when the illumination is broadband and spectrometers are used as detectors. Adapted with permission from Ref. 76 © Optica.

To measure $R_{d}$ and $T_{d}$, the overall reflected and transmitted light is collected by one or more integrating spheres, which integrate the light both in time and space. The reflectance and transmittance can be measured separately^2^^,^^4^^,^^14^ [Figs. 3(a) and 3(b) single-integrating sphere (IS)] or simultaneously^76^ [Fig. 3(c) double-integrating-sphere (DIS) system]. Common light sources used in IS and DIS systems are lasers^76^^,^^77^ and broadband halogen bulbs.^78^ The use of broadband laser-driven light sources has also been reported.^79^ As a collimated illumination beam is required in IS and DIS measurements, halogen light sources, and laser-driven light sources are usually used in combination with collimators. Common detectors are photodiodes^76^^,^^77^^,^^79^ for single-wavelength detection and spectrometers^78^^,^^79^ for broadband detection. Commercial spectrophotometers containing a built-in integrating sphere have also been used in IS measurements, albeit at a higher price point.^51^^,^^80^

$R_{d}$ and $T_{d}$ are the direct outputs of the computational AD method (see Sec. 3.1). Hence, the inverse AD (IAD) algorithm has been widely used to find $\mu_{a}$ and $\mu_{s}^{\prime }$. The IAD algorithm repeatedly guesses $\mu_{a}$ and $\mu_{s}^{\prime }$, calculates the corresponding $R_{d}$ and $T_{d}$ using the AD method, corrects $R_{d}$ and $T_{d}$ taking into account the integrating sphere geometries and properties, and compares the corrected values against the measured ones, until a match is made.^23^

The inverse Monte Carlo (IMC) method is another commonly used method for inversion.^2^^,^^14^ By setting a range of $\mu_{a}$ and $\mu_{s}^{\prime }$, a lookup table of $R_{d}$ and $T_{d}$ can be precomputed by MC simulations. When $R_{d}$ and $T_{d}$ are measured, the corresponding $\mu_{a}$ and $\mu_{s}^{\prime }$ can be found by interpolating the lookup table.

#### 4.1.2 Practical considerations

For both IS and DIS measurements, the integrating sphere properties should be well-known for accurate measurements, because sphere efficiency multiplies the signal detected. Properties, such as sphere size, port dimensions, and inner wall reflectance, as well as illumination angle and beam size, should be taken from the manufacturer or ideally characterized independently for each sphere. Light propagation in the DIS measurement is more complex than in the IS measurement due to “cross-talk” between the two spheres.^4^^,^^81^ For example, the light transmitted through the sample strikes on the transmittance sphere and becomes diffused, and the diffused light back-illuminates the sample; similarly, the light reflected from sample surface is diffused by the reflectance sphere and illuminates the sample again and so on [Fig. 3(c)]. A model has been implemented in the IAD algorithm to correct for these effects,^81^ and similar models can be implemented in the IMC method as well. Alternatively, in the IS measurement, the sphere efficiency can be corrected automatically if the measurement is conducted using a “comparison method,” where both the sample and reference are placed on the sphere at the same time and the sphere efficiency factor cancels out,^82^ or using a “normalization beam” that takes the integrating sphere inner wall reflectance as a reference and cancels out the sphere efficiency factor.^78^^,^^79^

Another factor that impacts the measured $R_{d}$ and $T_{d}$ in the steady state is the light loss at the sphere sample port edge, which leads to an overestimated $\mu_{a}$ if not taken into account in modeling.^83^ The light loss can be modeled by MC simulations that only consider photons re-emitting within the sample port. The more commonly used IAD algorithm has incorporated MC simulations to account for the light loss at the sample port edge.^81^

Uncertainty in thickness measurement also contributes toward measurement errors. Using a dial gauge micrometer, Lemaillet et al. measured thickness uncertainties in solid phantoms ranging from ±0.02 to ±0.18  mm depending on the phantom materials.^79^ These moderate errors in thickness measurements can translate into more substantial errors in the measured optical properties, and an overestimated thickness results in an underestimated $\mu_{a}$.

Uncertainties and errors in the IS and DIS measurements have been well documented.^84^^,^^85^ Accuracy in the range of 5%^76^ to 10%^77^ was reported for DIS measurements. Errors of 15% in $\mu_{a}$ and 5% in $\mu_{s}^{\prime }$ have been reported in the IS measurement.^84^ Nonetheless, when performing IS and DIS measurements on the same set of solid phantoms, it was found that the IS measurement can be more accurate than the DIS measurement.^79^ Moreover, by optimizing the IS setup, which is able to correct for the sphere efficiency, very low errors (3% in $\mu_{a}$ and 1% in $\mu_{s}^{\prime }$) have been reported.^78^ The inconsistency in reported accuracy may be explained by the choice of phantoms for characterization and how well the systems are calibrated.

Despite their calibration challenges, IS and DIS systems have been widely deployed to characterize *ex vivo* tissue samples as they are low cost and easy to set up. The in-house DIS system costs of the order of $15,000 and measures the reflectance and transmittance spectra within a couple of seconds (excluding the time for calibration measurements). Some commercial spectrophotometers have built-in integrating spheres for reflectance and transmittance measurements, and therefore, are also options although at a much higher cost. *Ex vivo* IS and DIS measurements have been used to create a library of $\mu_{a}$ and $\mu_{s}^{\prime }$ values for a variety of human and animal tissue types, including: skin, muscle, aorta, bladder, brain, breast, colon, liver, and lung, with additional characterizations on cancerous tissue samples.^2^^,^^14^

### 4.2 Time Domain Measurement Methods

TD techniques measure the tissue temporal response for an impulse illumination. A time resolved point detector records the temporal response. The conditions of the diffusive regime ($\rho \gg {\mathrm{mfp}}_{s}^{\prime }$) are obeyed by setting the source–detector separation at a couple of cm, or by translating to the photon propagation time t, so that measurements can be performed also at ρ=0 provided the traveled path of photons $\ell =\frac{c}{n}t\gg {\mathrm{mfp}}_{s}^{\prime }$ (see Sec. 2). The latter case is typically accomplished for t>50 to 100 ps, corresponding to an average of 10 to 20 effective isotropic scattering events for $\mu_{s}^{\prime }=10\;{\mathrm{cm}}^{-1}$ and g=0.9.

#### 4.2.1 Measurement methods

In the simplest setting of a semi-infinite medium and zero boundary condition—that is, making the harsh assumption that the fluence vanishes at the surface—diffusion theory shows that $R_{d}(\rho ,t)$ encodes both $\mu_{a}$ and $\mu_{s}^{\prime }$:^86^

$$R_{d}(\rho ,t)=\frac{z_{0}}{(4\pi Dv{)}^{3/2}}\cdot t^{-5/2}\cdot e^{-\frac{\rho^{2}+z_{0}^{2}}{4Dvt}}\cdot e^{-\mu_{a}vt}.$$ (3)

where $z_{0}=1/\mu_{s}^{\prime }$, v=c/n is the speed of light in the medium, and all other parameters are defined in Table 1. As in the diffusive regime, $\rho \gg 1/\mu_{s}^{\prime }$ and $\mu_{s}^{\prime }\gg \mu_{a}$, three terms can be identified in Eq. (3), that are: (i) $t^{-5/2}$, (ii) $e^{-\frac{3\rho^{2}\mu_{s}^{\prime }}{4vt}}$, and (iii) $e^{-\mu_{a}vt}$. The second term counterbalances the strong temporal decrease in signal due to the first term, causing a peak that is shifted to larger t upon increasing $\mu_{s}^{\prime }$. Conversely, the third term (Lambert–Beer) decreases the photon temporal survival probability depending on $\mu_{a}$. Therefore, $\mu_{s}^{\prime }$ and $\mu_{a}$ can be naturally disentangled in TD measurements, since they differentially affect the shape of $R_{d}(\rho ,t)$. Specifically, $\mu_{s}^{\prime }$ is related to the peak position, whereas $\mu_{a}$ relates to the asymptotic slope of the tail occurring at the order of ns.^1^^,^^86^^–^^88^ Further analysis on Eq. (3) shows that $\mu_{a}$ and $\mu_{s}^{\prime }$ can be expressed in closed-forms as functions of the mean and variance of $R_{d}(\rho ,t)$ for fast calculations.^89^ More refined boundary conditions can also be inserted—extrapolated boundary conditions assume the fluence vanishes at some distance from the surface^90^—and more complex geometries can be tackled, such as transmission through a slab^86^ or reflectance from a cylinder or a sphere.^19^

**Fig. 4:**
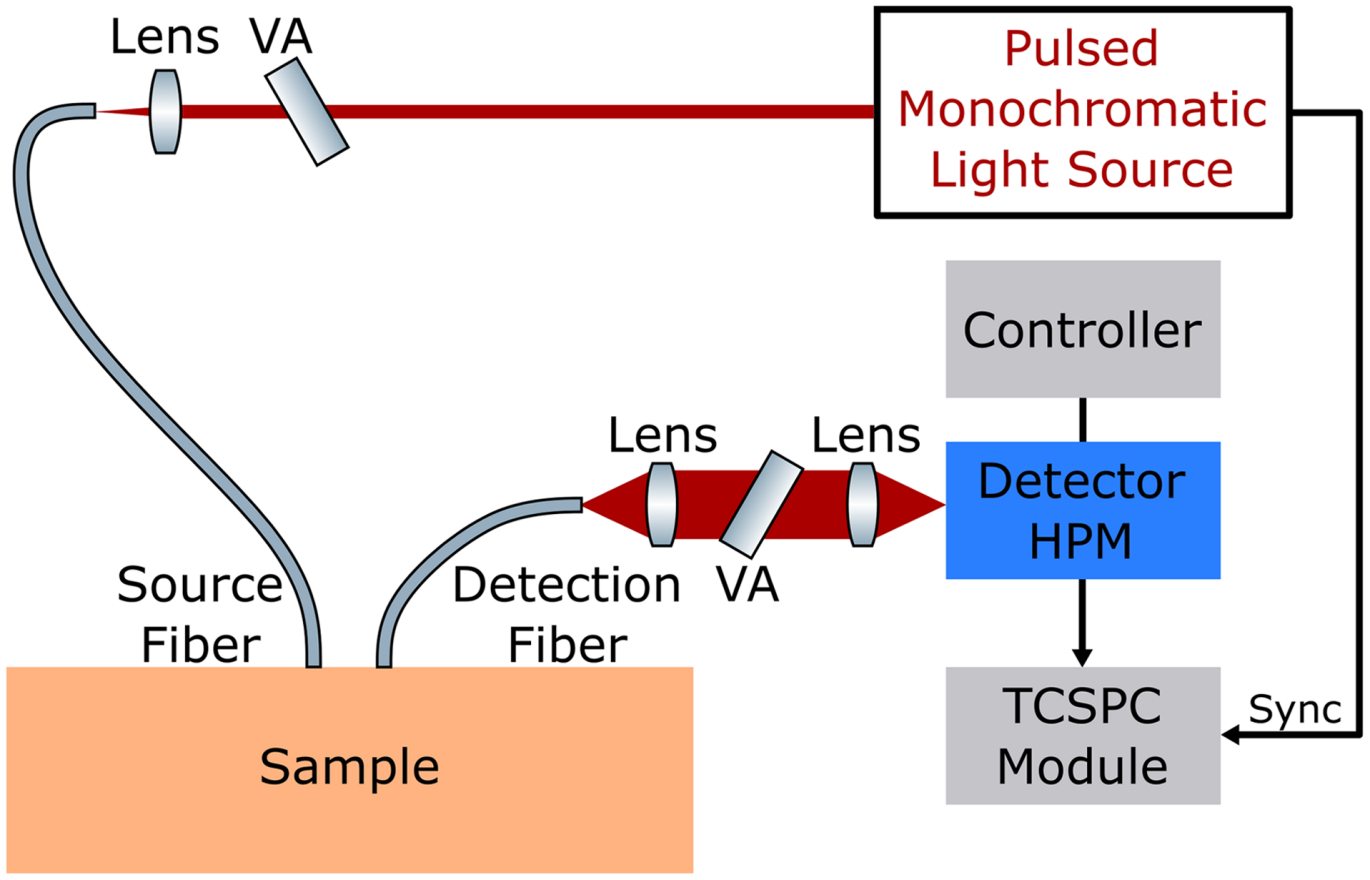
Schematic of a time-correlated single-photon counting (TCSPC)-based TD measurement system. A picosecond low-intensity pulsed monochromatic light source is used for illumination. Light is delivered and collected using fibers. The collected signal is detected by a hybrid photomultiplier detector and processed by a TCSPC module. The pulsed monochromatic illumination can be achieved by a picosecond low-intensity pulsed laser filtered by a tuneable filter to select the illumination wavelength. The intensity of illumination pulse is attenuated by a variable attenuator, so that no more than one photon reaches the detector for one pulse, and the detected photon has its arrival time detected, which follows the distribution of time-of-flight light spends in the sample. By repetitively delivering pulses and recording arrival time, the collected histogram is $R_{d}(\rho ,t)$ convolved with the instrument response function (IRF). Reproduced with permission from Ref. 88.

The TD measurement often requires a picosecond pulsed laser to generate the impulse illumination. On the detection side, in early implementations of the TD measurement, streak cameras^91^ and time-correlated single-photon counting (TCSPC) techniques^92^ were used for the time-resolved detection. Advances in the detection efficiency, time resolution, and compactness of the TCSPC technique make it the most common technique at present (Fig. 4), overtaking the less-sensitive, expensive, and bulky streak cameras.^1^^,^^93^^–^^95^

The measured temporal impulse response is, however, the convolution of $R_{d}(\rho ,t)$ with the instrument response function (IRF), so the shape of response changes. The IRF is the impulse response of the measurement system itself. State-of-the-art TCSPC-based TD measurement systems have IRFs around 100 ps in terms of full-width at half-maximum (FWHM).^4^^,^^87^^,^^96^^,^^97^ To take the IRF into account, $\mu_{a}$ and $\mu_{s}^{\prime }$ are found by fitting the measured signal with the convolution of the theoretical model with the IRF. For measurements at a short or even null ρ, the dynamic range of the system and the absence of long decay tails, even more than the FWHM, are crucial to avoid that the burst of early photons spread over late times, corrupting depth and absorption information.^98^^,^^99^ Quite noticeably, at null ρ, the photon temporal distribution becomes independent of $\mu_{s}^{\prime }$ (at least under the diffusion approximation), permitting an absorption estimate free of scattering contamination.^99^ Yet, this condition imposes harsh requirements on the measurement system and is usually avoided by adopting ρ≥2  cm instead, which is more tolerant with respect to IRF properties.

#### 4.2.2 Practical considerations

The salient advantage of TD measurement is its high accuracy, and the TD measurement is currently considered the “gold standard” of phantom characterizations. In a multicenter study of solid phantom standardization, 6 of 8 participating centers used TD measurement methods.^70^ Another multicenter study of liquid phantom standardization had 6 of 9 participants using TD measurement methods.^72^ Commercial reference phantoms^100^ are characterized with TD techniques to provide the true $\mu_{a}$ and $\mu_{s}^{\prime }$ for calibrations.^96^^,^^101^

A second advantage comes from the information-rich temporal response curve. The curve not only encodes $\mu_{a}$ and $\mu_{s}^{\prime }$ but also the depth information. Photons traveling further from the source arrive at a later time. Therefore, by exploiting the photon arrival time, it is possible to achieve depth- or in general spatial-sectioning,^95^^,^^102^ which can be applied to retrieve optical properties from layered tissues, such as the arm or the head.^103^

A third advantage is the independence of the surface properties when the medium is assessed with a homogeneous model relying only on the temporal shape of photon distribution. Indeed, a very thin (few μm) superficial layer, even highly absorbing, only affects the amplitude of a TD measurement. Therefore, any effect of skin pigmentation, optical contact, and laser instabilities can be canceled out.

Given the above advantages, TD measurements have been performed *in vivo* to characterize breast^104^ and brain tissues.^105^^,^^106^ Alternative approaches permitting to retrieve TD measurements without using pulsed lasers and TD detection are also proposed, such as the interferometric near-infrared spectroscopy system, which measures tissue temporal response and hence $\mu_{a}$ and $\mu_{s}^{\prime }$ from the interference spectrum generated by a wavelength-tuning coherent light source and its re-emitted light from tissue, with the additional benefit of retrieving blood flow from speckle fluctuations.^107^^,^^108^

Some issues must be considered in TD measurements. First, direct light due to light guiding effects at the interface between the skin and the probe can alter the measurement, yielding a sharp peak at early times. Soft black rubber covering the probe is useful to create light traps, whereas black plastics can still cause around 5% reflection. Transparent plates—e.g., glass or plastic plates—must be absolutely avoided due to light guiding effects, which cause a spurious spike at early times. Even if the thickness is as low as hundred microns, or the transparent foil is within the medium, light guiding can still disrupt the measurement. Though not visible, this effect is present also in continuous-wave measurements, probably with different impacts but still to be considered. Further, any side bands in the laser emission spectrum can lead to substantial underestimation of absorption for sharp spectral peaks (e.g., lipid peak at 930 nm). The reason is the strong change in the peak-to-sidebands signal ratio due to the exponential term in Eq. (3). Finally, system performances greatly affect the reliability of TD measurements. Temporal drifts cause errors in scattering and consequently absorption estimates but can be compensated by acquiring a reference pulse together with the signal.^109^ More than the FWHM of the IRF, the dynamic range and the presence of a long decay tail in the detector can hamper the chance to assess high absorption or to sense deep into the tissue.^98^ In general, the system performances must be tested directly on tissue phantoms, following established protocols, such as BIP for basic instrument performances,^110^ MEDPHOT for measurement on homogeneous media,^70^ NEUROPT for heterogeneous samples.^111^

From the hardware point of view, TD systems can be classified depending on the spectral coverage that can be (i) broadband (e.g., 600 to 1100 nm) with continuous sampling or (ii) discrete at a few wavelengths. In the first case, super-continuum pulsed lasers are adopted with very broad spectral emission.^88^^,^^112^ Streak-cameras,^113^ single-photon avalanche diode (SPAD) arrays, or multianode photomultiplier tubes (PMTs)^114^ can be adopted after a spectrometer for parallel detection, yet with much higher complexity with respect to classical charge-coupled devices (CCDs) used in the continuous-wave case, and with challenges to cope with huge spectral differences in signal intensity. A simpler path is to use a single detector with a TCSPC module, when the illumination is a wavelength scan achieved by the super-continuum source with a tuneable filter, which has the additional advantage of changing laser attenuation at each wavelength to cope with single-photon statistics (Fig. 4). The drawback of the scanning approach is longer acquisition time, typically 1 s per wavelength and so a few minutes per spectrum.^115^ Conversely, it is possible to operate at a few discrete wavelengths using gain-switched diode lasers, compatible with simultaneous and fast acquisition at the scale of ms per measurement. Therefore, fast tracking of hemodynamics and blood pulsatility at 10 to 100 Hz^116^ or fast spatial scanning in optical mammography at a rate of 40 pixel/s^104^ are feasible. Common detectors are PMTs, but more compact and robust SPAD detectors are emerging.^95^^,^^117^ One limitation of using SPADs is their small active area, detecting fewer photons and lowering the signal-to-noise ratio (SNR).^95^^,^^118^ A silicon photomultiplier (SiPM), which is a high-density matrix of SPADs connected in parallel, has a larger active area for more efficient detection. Being low-cost and compact as well, SiPMs are becoming a new solution to TD detection.^95^^,^^96^^,^^118^^–^^121^

By far the majority of TD systems are laboratory prototypes, with a few commercial products available for biomedical applications.^95^ The limited commercialization of TD spectroscopy measurements can be attributed to their high costs, limited scalability, and complex designs,^4^^,^^95^^,^^122^ which are inevitable for the current technology to meet the required high time resolution. The internal cost estimate of a TCSPC-based TD system for broadband spectroscopy is of the order of $200,000. Systems operated at discrete wavelengths have a lower size and cost, in the order of $50,000, with some commercial devices already on the market, such as NIRSBOX by PIONIRS,^123^ operated at two wavelengths for the measurement of tissue oxygenation and hemodynamics.^124^

### 4.3 Time-Frequency Domain Measurement Methods

Time FD is the FT of the time domain, so FD techniques measure the tissue frequency response for an intensity-modulated point illumination. Point detectors are placed some distance (ρ at the order of a few cm) away and record the amplitude (M or amplitude modulation equivalently in some measurements) and phase (ϕ) of the reflected light.

#### 4.3.1 Measurement methods

The derivations of M and ϕ as functions of $\mu_{a}$ and $\mu_{s}^{\prime }$ using diffusion approximation are well documented.^1^^,^^90^^,^^125^^,^^126^ The expressions of M and ϕ are complicated and encode both $\mu_{a}$ and $\mu_{s}^{\prime }$, which requires a fitting process on M and ϕ to extract $\mu_{a}$ and $\mu_{s}^{\prime }$. M and ϕ depend on both ρ and the frequency of light intensity modulation. Therefore, M and ϕ can be measured with single-distance multifrequency or multidistance single-frequency methods, to generate a dataset for fitting. Alternatively, after further approximations, it has been shown that the slopes of ϕ and $\ln(\rho^{2}M)$ with respect to ρ are functions of $\mu_{a}$ and $\mu_{s}^{\prime }$, and closed-form expressions of $\mu_{a}$ and $\mu_{s}^{\prime }$ exist.^1^^,^^127^ To obtain the slopes with respect to ρ, this method is intrinsically multidistance, as signals have to be measured at several different ρ. Due to limitations of diffusion theory (see Sec. 3.1), diffusion models can give good approximation only up to 1 GHz.^90^ Although most of the FD measurements have used diffusion approximation models, IMC is now becoming the standard for FD data analysis.^128^

**Fig. 5:**
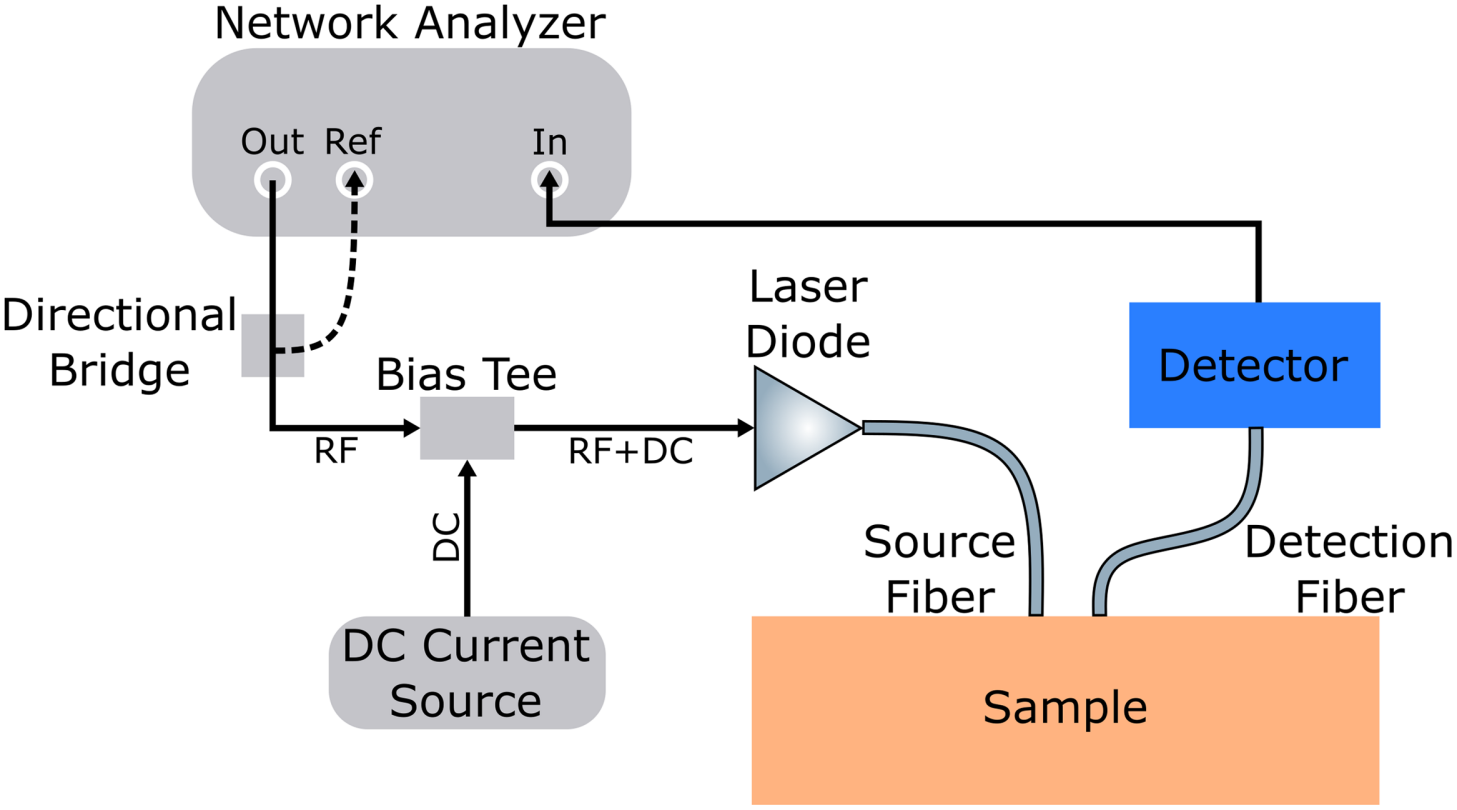
Schematic of a FD single-distance multifrequency measurement system based on a network analyzer. The network analyzer generates RF current, a fraction of which is fed back through a directional bridge to the reference channel of the network analyzer to determine the amplitude and phase of the reference signal. The RF signal is superimposed with the DC signal by a bias tee, in order to modulate the laser diode. Light from the laser diode is delivered and collected using fibers. The collected light signal is converted to RF signal by a detector and is sent back to the network analyzer to extract its amplitude and phase. More laser diodes can be included in the schematic, connected in parallel with switches, for more illumination wavelengths. Adapted with permission from Ref. 130 © Optica.

The FD measurement needs an intensity-modulated point light source, e.g., a laser diode, which is usually modulated by an RF driver,^127^ a network analyzer^125^^,^^126^^,^^129^^–^^131^ (Fig. 5), or recently low-cost and compact direct digital synthesizers.^128^^,^^132^^–^^134^ As noted above, the measurement can be performed either in multidistance single-frequency or single-distance multifrequency modes. At the detector side, PMTs^135^ and, more commonly, compact and lower-cost avalanche photodiodes (APDs)^125^^,^^128^^,^^132^^,^^134^^–^^136^ are used to amplify the weak detection signal. Recently, the use of SiPMs in FD systems is being investigated, because compared with conventional APDs, SiPMs can have a higher SNR at a lower reverse-bias voltage.^135^^,^^137^ The amplitude and phase of the detected signal are then resolved by homodyne detection^138^^,^^139^ or more commonly heterodyne detection,^1^^,^^125^^,^^127^ which can also be achieved by a network analyzer. Recent advances in electronics allow one to digitally sample the detected signal, followed by postprocessing, such as fast FT or Goertzel algorithm, to extract the phase and amplitude.^134^^,^^140^

#### 4.3.2 Practical considerations

An obvious practical concern with FD methods is the selection of modulation frequency.^141^ Too low, a frequency would lead to a small shift in ϕ, which is difficult to detect; too high, a frequency would attenuate the amplitude substantially for detection, in addition to the limit set by the diffusion models. As a result, moderate frequency, of the order of 100 MHz, is often used. The choice of illumination wavelength is limited by measurement sensitivities to amplitude and phase. FD measurements are substantially less sensitive to the tissue optical properties common in the wavelength range longer than 1000 nm compared to the NIR-I window,^142^ in addition to the potential hardware limitations in light modulation and detection at long wavelengths.^143^ Another practical concern is calibration, as the phase and gain of the measurement system should be carefully calibrated using a reference phantom^125^^,^^127^ or canceled in measurements through special arrangements of sources and detectors.^125^^,^^144^

Low measurement errors have been reported by well-calibrated FD measurement systems. For example, ±5% error in $\mu_{a}$ and ±3% error in $\mu_{s}^{\prime }$ were reported on Intralipid phantoms using multifrequency FD measurement, but the error increased substantially for a low absorbing sample ($\mu_{a}<0.001\;{\mathrm{mm}}^{-1}$).^125^ In addition to absolute optical property characterizations, the FD measurement provides information about amplitude and phase. The relative changes in amplitude and phase measured on brains have been related to response to stimulus, and FD techniques can monitor these fast optical signal at a time scale of 100 ms.^141^ With these promising accuracies, the FD measurement has been applied to characterize phantoms for system calibration and testing.^145^ *In vivo* FD measurements include those on breast^126^^,^^129^^–^^131^^,^^143^ and brain.^127^^,^^141^

As many FD measurement devices are based on network analyzers for modulation and demodulation, they have large footprints and high costs (∼$30,000 to $75,000).^126^^,^^136^^,^^146^ Furthermore, the data acquisition is relatively slow. Using a network analyzer, a single-distance multifrequency measurement sweeping 401 modulation frequencies requires 1 s for a single wavelength.^146^ Shorter acquisition time can be achieved using fewer frequencies or the single-frequency multidistance measurement but at the cost of potentially larger measurement errors.^122^ With advanced hardware, faster measurements are possible with the use of wavelength multiplexing, frequency division multiplexing, and faster electronics.^122^^,^^132^^–^^134^^,^^136^^,^^140^^,^^146^ Recent advances in digital signal generation and detection have enabled digital electronics to replace the expensive and bulky network analyzers, thereby reducing implementation costs,^137^^,^^147^ increasing measurement speed,^133^^,^^136^ and improving scalability.^133^^,^^137^ For example, acquisition time as short as 27  μs for a single-wavelength single-frequency measurement is achieved by a digital FD system,^133^ and cost as low as $600 for a single-wavelength device becomes feasible with digital electronics.^137^ A commercial dual-wavelength FD device (OxiplexTS, ISS)^148^ is now marketed based on the multidistance single-frequency (110 MHz) measurement, to measure $\mu_{a}$ and $\mu_{s}^{\prime }$ and the derived hemoglobin concentrations for monitoring oxygen saturation in brain and muscle.

### 4.4 Spatial Domain Measurement Methods

SD techniques measure the tissue spatial response for a continuous-wave point illumination. The detection of intensity is conducted at several known distances (ρ) away from the point source. Similar to TD and FD measurements, the average of detector distances is of the order of a few cm.

#### 4.4.1 Measurement methods

Assuming a semi-infinite homogeneous medium, $R_{d}(\rho )$ can be derived from diffusion theory as^149^

$$R_{d}(\rho )=\frac{a^{\prime }}{4\pi }[z_{0}(\mu_{\mathrm{eff}}+\frac{1}{r_{1}})\frac{e^{-\mu_{\mathrm{eff}}r_{1}}}{r_{1}^{2}}+(z_{0}+2z_{b})(\mu_{\mathrm{eff}}+\frac{1}{r_{2}})\frac{e^{-\mu_{\mathrm{eff}}r_{2}}}{r_{2}^{2}}],$$ (4)

where $z_{0}=1/\mu_{s}^{\prime }$, $z_{b}=2\mathrm{AD}$, $r_{1}=\sqrt{z_{0}^{2}+\rho^{2}}$, $r_{2}=\sqrt{(z_{0}+2z_{b}{)}^{2}+\rho^{2}}$, and all other parameters are defined in Table 1. Thus $\mu_{a}$ and $\mu_{s}^{\prime }$ can be found by fitting the absolute measurement of $R_{d}(\rho )$ with Eq. (4). When ρ is sufficiently large, Eq. (4) can be further simplified and leads to

$$\frac{d\;\ln(R_{d}(\rho ))}{d\rho }\approx -\mu_{\mathrm{eff}}-\frac{2}{\rho }.$$ (5)

Therefore, it is clear that the relative shape of $R_{d}(\rho )$ only depends on $\mu_{\mathrm{eff}}$, and there is no unique solution of $\mu_{a}$ and $\mu_{s}^{\prime }$ from the relative $R_{d}(\rho )$ measurement.

**Fig. 6:**
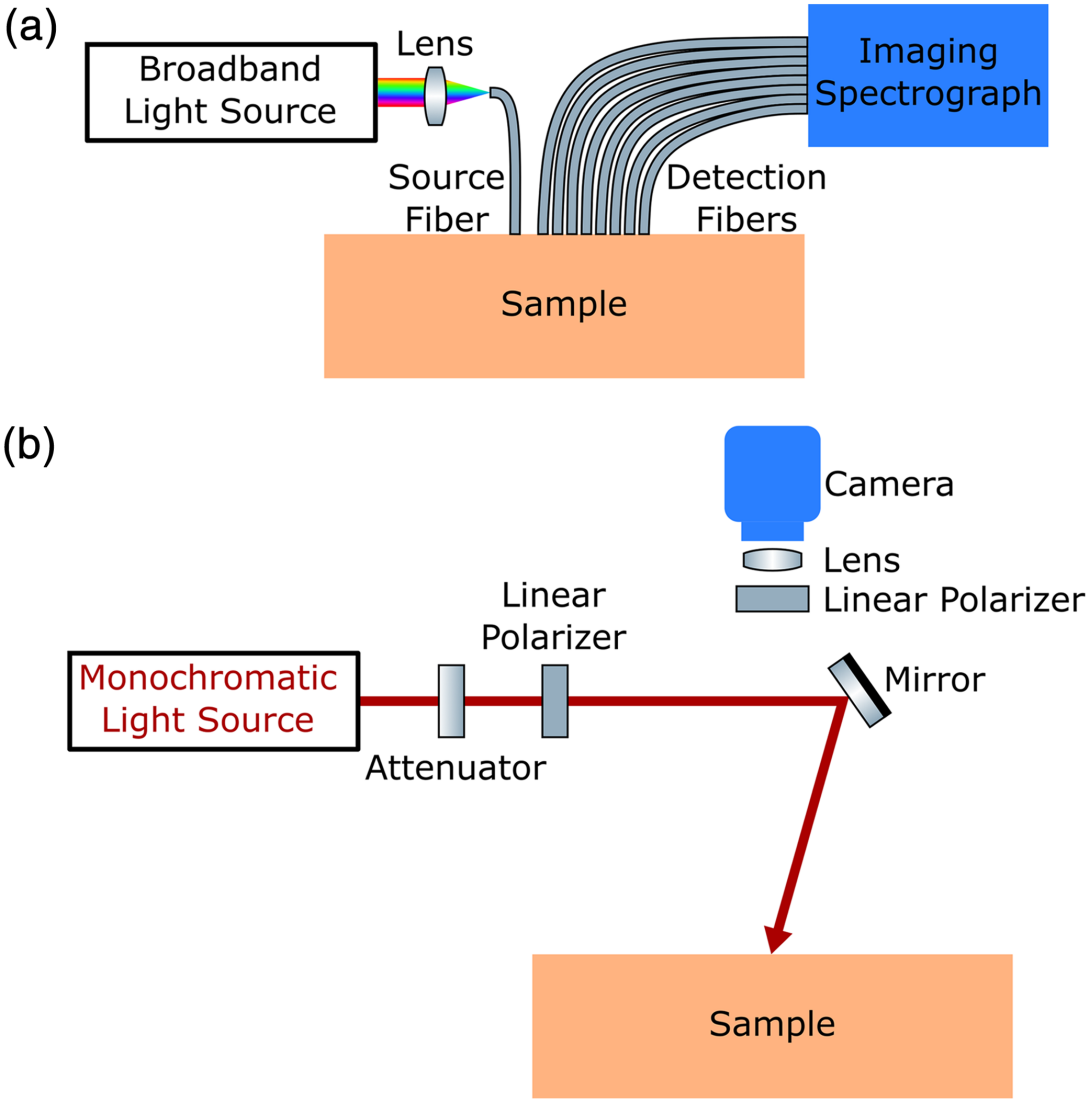
Schematics of spatial domain (SD) measurement systems. The multidistance measurement in (a) uses a broadband light source delivered by fiber optics to the sample, and $R_{d}(\rho )$ is spatially sampled by eight fibers and is spectrally resolved by an imaging spectrograph. (b) A monochromatic light source is used to illuminate the sample, and $R_{d}(\rho )$ is spatially resolved by a wide-field camera to detect pixel-wisely. Cross polarization can be implemented by linear polarizers, to minimize specular reflection. Adapted with permission from Ref. 151 © Optica.

To spatially resolve $R_{d}(\rho )$, SD measurement systems either use an array of detection fibers^149^^,^^150^ [Fig. 6(a)] or a camera^151^^–^^153^ [Fig. 6(b)]. Figure 6(a) shows a broadband detection method, detecting $R_{d}(\rho )$ spectra and hence measuring $\mu_{a}$ and $\mu_{s}^{\prime }$ spectra. A commonly used broadband light source is a halogen bulb.^150^^,^^154^ At the detection end, a spectrograph is often chosen, resolving the spectral output from the fiber array in a single device, rather than using multiple individual spectrometers.^150^^,^^154^ For a monochrome camera-based system like depicted in Fig. 6(b), single-wavelength measurements are taken using a monochromatic light source, such as a laser diode^151^^,^^152^ or a broadband light source filtered by a monochromator.^153^ Wavelength scans take a longer time than the broadband detection methods if many wavelength points are included to reconstruct a spectrum.

The absolute measurement of $R_{d}(\rho )$ needs careful calibration and characterization.^151^^,^^155^ In contrast, relative measurements are easier to perform.^150^^,^^153^

Diffusion models break down near the source (see Sec. 3.1), which leads to substantial errors in the fitting process of Eq. (4) if short detector distances are included. The IMC method has no such problems and has been used with the SD measurement.^151^^,^^156^

#### 4.4.2 Practical considerations

As mentioned above, despite the ease of instrumentation, relative measurements do not lead to a unique solution of $\mu_{a}$ and $\mu_{s}^{\prime }$. Several methods have been proposed for this problem. Doornbos et al.^150^ introduced constraints in the fitted scaling factors of relative $R_{d}(\rho )$ to be within little variation, as well as the shape of $\mu_{s}^{\prime }$ spectrum to follow Mie scattering, in order to stabilize the fitting process. Suzuki et al.^157^ discarded the absolute quantification of optical properties and only measured the relative $\mu_{a}$ and its derived oxygen saturation from the slope of $\ln(R_{d}(\rho ))$ [Eq. (5)], assuming a linearly decreasing $\mu_{s}^{\prime }$ spectrum. The use of relative $\mu_{a}$ is exploited in some commercial continuous-wave spatially resolved near-infrared spectroscopy (NIRS) devices,^158^ such as the three-wavelength oxygenation monitor (NIRO-200NX, Hamamatsu).^159^ Combining the constraints in $\mu_{a}$ and $\mu_{s}^{\prime }$ spectra shapes and the use of $\ln(R_{d}(\rho ))$ slope is also implemented for oxygenation measurements.^154^

The accuracy of SD measurement is moderate. Compared to other measurement methods, 5% to 10% deviations in optical property estimates were observed,^149^ but higher errors (20%^153^ to 40%^150^) have also been reported. Additional measurements on total diffuse reflectance were performed together with the SD measurement, but estimation errors remained at 10%.^152^^,^^160^ Neural networks trained on MC simulations were attempted, which still possessed a 14% error in $\mu_{a}$ estimate.^151^ For the relative $\mu_{a}$ measurement in spatially resolved NIRS, the derived oxygen saturation shows good consistency with the reference measurement obtained by a blood gas analyzer.^157^

The simple and low-cost (<$15,000) setup is an advantage for *in vivo* SD measurements. Short data acquisition time is an additional benefit for real-time applications, and the measurement can be made at a time scale of seconds.^150^^,^^154^^,^^155^ *In vivo* absolute characterizations of $\mu_{a}$ and $\mu_{s}^{\prime }$ have been performed on human skin,^150^^,^^152^^,^^156^ healthy and malignant breast tissues,^161^ as well as esophageal tissue through endoscopic spatially resolved reflectometry.^162^ Spatially resolved NIRS has found clinical applications in oxygenation monitoring of tissues, such as brain and muscle.^159^

### 4.5 Spatial Frequency Domain Measurement Methods

Spatial FD measurements had remained a theoretical construct as the FT of spatial domain for a long time, until the invention of spatial frequency domain imaging (SFDI).^145^ SFDI measures the tissue spatial frequency response for a spatially structured continuous-wave plane illumination, recording $R_{d}(k)$ as a function of spatial frequency k.

#### 4.5.1 Measurement methods

Assuming a semi-infinite homogeneous linear medium with spatially modulated illumination source, diffusion theory shows that^145^

$$R_{d}(k)=\frac{3a^{\prime }}{\left(1+\frac{\mu_{\mathrm{eff}}^{\prime }}{\mu_{t}^{\prime }})\cdot (3+2A\cdot \frac{\mu_{\mathrm{eff}}^{\prime }}{\mu_{t}^{\prime }}\right)},$$ (6)

where $\mu_{\mathrm{eff}}^{\prime }=\sqrt{\mu_{\mathrm{eff}}^{2}+k_{x}^{2}+k_{y}^{2}}$, and $k_{x}$ and $k_{y}$ are the angular spatial frequencies in x and y directions, respectively, with $k^{2}=k_{x}^{2}+k_{y}^{2}$. When k=0, Eq. (6) reduces to Eq. (2) and only depends on $a^{\prime }$. At higher k, $a^{\prime }$ is no longer the only source of optical contrast, showing the potential to separate $\mu_{a}$ and $\mu_{s}^{\prime }$. The nature of tissue low-pass spatial filtering can be observed from Eq. (6): as k increases, $\mu_{\mathrm{eff}}^{\prime }$ increases and $R_{d}(k)$ decreases. In addition to Eq. (6), other models of $R_{d}(k)$ using diffusion approximation, such as the FT of Eq. (4) for $R_{d}(\rho )$, reveal the same low-pass nature and coincide with Eq. (6) at low k.^163^

**Fig. 7:**
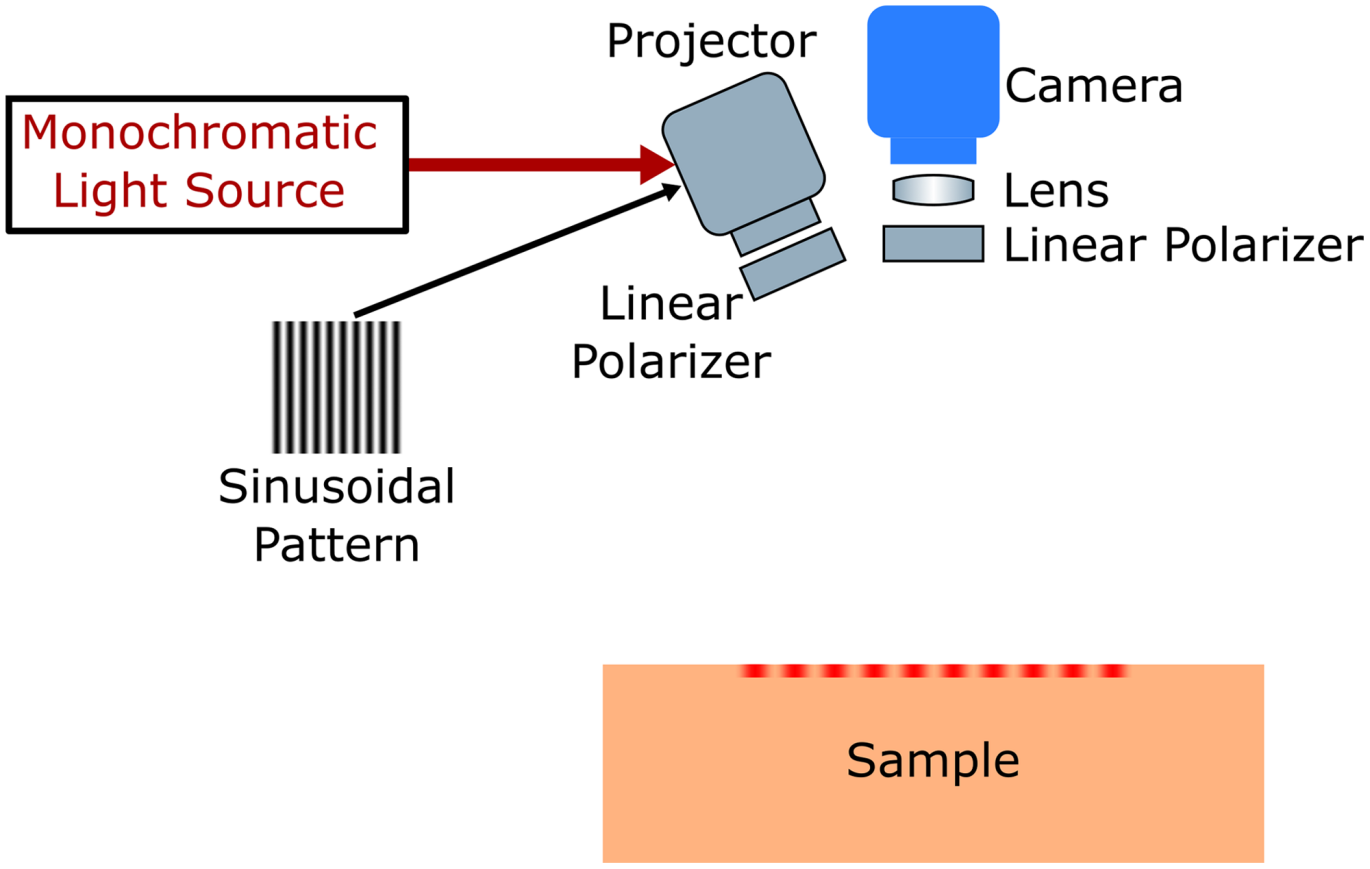
Schematic of the spatial frequency domain imaging (SFDI) system. A monochromatic sinusoidal pattern is projected onto the sample by a projector. The reflected light is detected by a camera. Cross polarization is implemented by linear polarizers to minimize specular reflection. Reproduced with permission from Refs. 73 and 164.

A typical SFDI system is Fig. 7. Commonly used (quasi-)monochromatic light sources are discrete LEDs,^164^^–^^166^ laser diodes,^167^^,^^168^ broadband lamps in combination with filters,^145^ and wavelength-tunable lasers,^169^ depending on the number of wavelengths being investigated and the system size, complexity, and cost.^73^ Plane illumination of sinusoidal pattern is commonly realized by spatial light modulators, such as digital micromirror devices^73^^,^^164^ or simply commercial projectors.^73^^,^^145^ The pattern reflected by the tissue is captured by a monochrome-camera. To minimize specular reflection, cross polarization is frequently implemented.^164^ The reflected light needs to be demodulated and calibrated to obtain $R_{d}(k)$.

Single-pixel demodulation methods are the standard ways to extract the response, which involves illuminating the sinusoidal pattern three times at the same spatial frequency but at three equally spaced (120 deg) phases.^73^^,^^145^ The three captured images can be manipulated so that both the AC (at k≠0) and DC (at k=0) responses are obtained at the resolution of camera.^73^^,^^145^ The fact that DC response can be calculated from the AC pattern is because the AC illumination is positively biased with a DC constant to achieve non-negative intensities. Therefore, measurements at two spatial frequencies can be completed with three images.

Alternatively, multipixel demodulation methods consider neighboring pixels to extract the response.^73^ One implementation of this method is single snapshot of optical properties (SSOP), which projects a single-sinusoidal pattern only.^170^ The captured image is analyzed in FD by performing FT, and the DC and AC responses are extracted by low-pass and band-pass filtering the Fourier signal.^168^^,^^170^ SSOP method only requires one image, which is more suitable for real-time applications, but at the cost of degraded image quality.^168^^,^^170^

From Eq. (6), $R_{d}(k)$ measured at two spatial frequencies is sufficient to separate $\mu_{a}$ and $\mu_{s}^{\prime }$. By modeling $R_{d}(k)$ for a range of $\mu_{a}$ and $\mu_{s}^{\prime }$ at two spatial frequencies, a lookup table can be constructed. $\mu_{a}$ and $\mu_{s}^{\prime }$ can then be solved by interpolating the lookup table. ^145^ The lookup table generated by the IMC method is also applicable to SFDI, with an additional benefit of improved accuracy.

#### 4.5.2 Practical considerations

Calibration of SFDI is needed to remove the IRF. As SFDI is in the FD, the convolution of the system point spread function in the real spatial domain becomes a multiplication of the system modulation transfer function. Therefore, the system’s IRF can be measured and removed through a division of the measured response by the theoretical $R_{d}(k)$ of a calibration phantom.^73^^,^^145^ Both the DC and AC responses have to be demodulated and calibrated, and $R_{d}(k)$ at two spatial frequencies are then readily measured to extract $\mu_{a}$ and $\mu_{s}^{\prime }$.

The AC frequency is usually set to a value between 0.1 and $0.2\;{\mathrm{mm}}^{-1}$.^164^ For the diffusion models to be valid, the spatial frequency should be much lower than $\mu_{t}^{\prime }$.^73^^,^^145^ A high spatial frequency will also lead to a small $R_{d}(k)$ given the low-pass filtering nature of tissue, which degrades the signal-to-noise ratio for detection. Too low a frequency, however, will give a spatial period larger than the area of illumination and detection.^145^ The impact of frequency selection on the measurement uncertainty has been carefully considered elsewhere.^171^

Optical properties estimated by SFDI are generally in good agreement with other standard measurement methods. Compared to the FD measurement, small deviations in $\mu_{a}$ (6%) and $\mu_{s}^{\prime }$ (3%) estimates on flat phantoms were reported, albeit with an overestimation in $\mu_{a}$ for low absorbing samples ($\mu_{a}=0.002\;{\mathrm{mm}}^{-1}$).^145^ Errors in optical property estimates increase for nonflat samples. Nonflat surfaces distort the projected pattern and the reflected light intensity changes with height variations and surface angles.^73^^,^^166^^,^^172^ Errors in $\mu_{a}$ as high as 10% ${\mathrm{cm}}^{-1}$ per height variation and 86% at 40 deg tilt have been observed.^172^ To correct for these errors, the sample surface profile can be reconstructed using phase profilometry that analyzes the deformed spatial frequency pattern. With the surface profile known, the reflected light intensity can be corrected according to the height and angle derived from the profile, hence the correction of estimated $\mu_{a}$ and $\mu_{s}^{\prime }$.^166^^,^^172^

The salient advantage of SFDI is the noncontact wide-field characterization generating 2D $\mu_{a}$ and $\mu_{s}^{\prime }$ maps. Spatial resolutions of 0.3 mm in $\mu_{a}$ map and 0.05 mm in $\mu_{s}^{\prime }$ map have been reported.^145^ Maps of oxygenation and hemoglobin concentrations can be subsequently derived from the $\mu_{a}$ map.^167^ In addition, SFDI systems can be implemented at low costs (e.g., OpenSFDI at $4717^164^) and small footprints, which makes the technique more accessible. As a result, SFDI has been deployed in many applications, such as chronic wound examinations, burn imaging, surgical guidance, cancer detection, skin characterization, and in neuroscience.^73^ A commercial SFDI device (Clarifi, Modulim) is also available to visualize oxygenation and perfusion in lower limb complications.^173^ Furthermore, SFDI can be used to perform depth-sensitive imaging or tomography,^73^^,^^174^^,^^175^ because sinusoidal patterns at different spatial frequencies are equivalent to different source–detector separations interrogating the sample at different depths.^176^ However, this also makes SFDI susceptible to partial volume errors since two disparate spatial frequencies are required to extract optical properties.^171^ More discussions on partial volume errors are in Sec. 5.

For a fair comparison of data acquisition speed, the fact that, among all the estimation methods discussed, only SFDI can produce optical property estimation maps without spatial scanning should be noted. For SFDI, the data acquisition speed is limited by the camera exposure time, the number of images to capture, and the number of wavelengths. Typically, exposure time at 100 ms^164^ and three images per wavelength are required for the standard single-pixel demodulation method, and additional images may be needed for surface profilometry. Taking a series of images slows down the measurement and may introduce motion artifacts.^73^ To acquire data faster and enable real-time measurements, high-speed cameras have been used at shorter exposure times^177^ and the SSOP method has been applied to reduce the number of images.^178^ In addition, the pattern switching time of the projector also limits the acquisition time. It has been shown that binary square-wave patterns can be switched at a higher rate than the conventional gray-scale sinusoidal patterns, while retaining comparable accuracy in optical property estimations.^177^^,^^179^

### 4.6 Comparison of Estimation Methods

Table 2 compares all the estimation methods described above in terms of their measurement capability, inversion method, accuracy, acquisition speed, system size and cost, as well as whether commercial products and open-source guides are available. In Table 2, only a qualitative comparison of accuracy is provided, given the often inconsistent numerical accuracy reported for each estimation method. The inconsistency in reported accuracy may be explained by the choice of phantoms for characterization and how well the systems are calibrated. There is an unmet need for standard phantoms to calibrate and characterize measurement systems, as different instruments are currently being compared against different standards, frequently either Intralipid-based phantoms, for which the optical properties can be found in the literature, or phantoms that have been characterized by TD or FD measurements, which are usually considered as highly accurate “gold-standard” methods. In addition, most instruments show higher accuracy for a certain range of $\mu_{a}$ and $\mu_{s}^{\prime }$ over other ranges. For example, FD and SFDI measurements have higher errors for low absorbing samples (see Secs. 4.3 and 4.5). As a result, reported accuracies may be biased when testing the instrument with phantoms that only cover a subrange of optical properties or selecting the range where the instrument performs most reliably.

**Table 2:** Comparison of the estimation methods described in Secs. 4.1–4.5.

Domain	Steady state	TD	Time FD	Spatial domain	Spatial FD
Technique	Single-integrating-sphere, double-integrating-sphere systems	Time resolved spectroscopy, also known as, TD diffuse optics	FD photon migration, also known as, FD diffuse optics	Spatially resolved spectroscopy	Spatial FD imaging
Measurement sample	*Ex vivo* only	*Ex vivo* and *in vivo*	*Ex vivo* and *in vivo*	*Ex vivo* and *in vivo*	*Ex vivo* and *in vivo*
Measurement geometry	Reflection and transmission	Reflection or transmission	Reflection	Reflection	Reflection
Inversion methods	Inverse adding-doubling, inverse Monte Carlo	Diffusion model, inverse Monte Carlo	Diffusion models, inverse Monte Carlo	Diffusion models, inverse Monte Carlo	Diffusion model, inverse Monte Carlo
Accuracy	Good	Very high	High	Moderate	High
Acquisition time	short ∼s/spectrum	Moderate^a^–long^b^ ∼0.1 to 1 s/wavelength	Short^c^–long^d^ ∼μs-s /wavelength	short^e^ ∼s/spectrum	moderate^f^ ∼0.1 to 0.3 s/wavelength
System footprint	Small	Moderate^a^–very large^b^	Small^g^–large^h^	Small	Small
System cost^i^	Low <$15,000	High^a^–very high^b^ ∼$50,000 to $200,000	Low^g^–high^h^ <$1000 to ∼$50,000	Low <$15,000	Low <$10,000
Commercial product example	Spectrophotometer, Perkin Elmer, SphereSpectro, Gigahertz-Optik	NIRSBOX, PIONIRS	OxiplexTS, ISS	NIRO-200NX, Hamamatsu	Clarifi, Modulim
How-to guide	81	—	—	—	164

a Discrete-wavelength TD devices.

b Broadband TD spectroscopy.

c Single-distance single-frequency FD measurement based on digital electronics.

d Single-distance multifrequency FD measurement based on a 401-frequency network analyzer.

e SD measurement using the fiber-array-based system, e.g., Fig. 6(a).

f SFDI using the SSOP or single-pixel demodulation methods.

g FD measurement systems based on digital electronics.

h FD measurement systems based on network analyzers.

i For laboratory prototypes.

Table 2 can be used to guide the selection of estimation methods for a certain application. For applications that need high accuracy, such as characterizing calibration phantoms, the TD measurement is preferred. For low-cost applications, which still need good accuracy, SFDI should be considered, and the steady-state measurement can also be chosen if the sample is *ex vivo* tissue or a phantom slab. SFDI is also suitable for imaging applications where 2D maps of $\mu_{a}$ and $\mu_{s}^{\prime }$ are required. Although 2D images of $\mu_{a}$ and $\mu_{s}^{\prime }$ maps can also be obtained using TD or FD techniques, these measurements involve either spatial scanning over the tissue or complex arrays of many source–detector pairs,^104^^,^^117^^,^^122^ which are more complicated to build and operate than the SFDI setup. For real-time qualitative monitoring of tissue optical properties where accurate absolute estimation is not the primary goal, the SD measurement can be a suitable candidate.

## 5 Limitations

There are some common limitations shared by all or subsets of the measurement methods described above. Many of the measurement methods make contact with the sample during the measurement, either with integrating spheres or fiber probes (see Secs. 4.1–4.4). The applied pressure can change the measured optical properties.^180^^–^^182^ The issue of pressure-induced optical property change highlights the importance of noncontact measurement methods, such as SFDI. Also, light sneaking in-between skin and probe—particularly for a sleek surface—can produce subtle contamination.

Another limitation is the assumption of sample homogeneity. Applying the homogeneous model to analyze inhomogeneous tissues, such as layered media like skin, results in partial volume errors. The steady-state measurement, FD multidistance single-frequency measurement, and SD measurement assume the sample to be homogeneous and report the averaged optical properties over the area of interrogation. The TD and FD single-distance multifrequency measurements typically have only one source–detector pair. As the light travels along the paths between the source and detector, any inhomogeneities in $\mu_{a}$ and $\mu_{s}^{\prime }$ along the paths are averaged out. SFDI, as mentioned in Sec. 4.5, is also susceptible to partial volume errors due to the used two disparate spatial frequencies. Exploiting the spatial-sectioning in TD measurements, more advanced multilayer models, or tomography, can mitigate this problem. 3D reconstructions of optical property distributions can be more commonly achieved by diffuse optical tomography,^1^^,^^4^^,^^183^^,^^184^ but also at a low resolution.^4^^,^^185^ An alternative solution to sampling heterogeneity is to only measure the local optical properties using very short source–detector separations.^186^ However, if the source–detector separation is comparable to ${\mathrm{mfp}}_{s}^{\prime }$, the diffusion approximation is no longer applicable and using $\mu_{a}$ and $\mu_{s}^{\prime }$ to quantify light propagation is not sufficient.

The limited access to affordable standard phantoms hinders accurate calibrations and characterizations of the low-cost measurement methods. The MEDPHOT protocol specifies a standard solid phantom-based protocol to measure $\mu_{a}$ and $\mu_{s}^{\prime }$ in homogeneous media^70^ and has been applied by a wide range of institutions, but the commercial phantom sets manufactured based on the MEDPHOT protocol are costly and have limited accessibility. Commercial solid phantoms on the current market usually have limited availability with few off-the-shelf products and are made out of various material types and characterized by different measurement setups.^96^^,^^100^^,^^101^^,^^187^^,^^188^ Multicenter studies on the same set of solid phantoms using different instrumental setups revealed deviations of up to 15% for measurements of $\mu_{a}$ and $\mu_{s}^{\prime }$.^70^^,^^97^ Phantoms that offer traceability to the International System of Units would enable comparable measurements across instruments, methodologies, times, and locations, but there are only limited examples in biophotonics community.^189^^,^^190^ Liquid phantoms based on Intralipid-20% and India ink have been proposed as easy obtainable references by many and have shown high measurement consistency (2%) in multicenter studies.^71^^,^^72^ However, liquid phantoms are not as robust, durable, and simple-to-handle as solid phantoms and limited in applicability. Moreover, this approach can be highly sensitive to changes in the manufacturing process.^35^ As biological tissues come with high intrinsic heterogeneity and cross-sample variability, a higher measurement accuracy is desirable, requiring better access to standard phantoms and stringent testing methodologies that are thoroughly followed. Additionally, the careful implementation, documentation, and understanding of the characterization measurement and its associated uncertainties are vital to ensure its reproducibility and to maximize its value.^35^

The choice of interrogating wavelengths limits the types of chromophore that can be detected and the penetration depth that can be achieved. Conventionally, the NIR-I window targeting on oxyhemoglobin and deoxyhemoglobin is implemented in most systems. Extending to short-wavelength infrared (SWIR) wavelengths (1000 to 2500 nm) enables the detection of water, lipids, and collagen. In principle, the decreased $\mu_{s}^{\prime }$ should lead to deeper SWIR light penetration,^191^^,^^192^ yet this is counterbalanced by higher $\mu_{a}$ of water and lipids, which limits the maximum photon pathlength, and therefore, the effective probed depth. As a result, optimal illumination wavelengths have to be identified to trade-off between chromophore types, absorption, and scattering.^165^ Measurements at SWIR wavelengths have been done mostly in steady state,^191^^,^^193^^–^^195^ but the number of studies of SWIR light in other measurement domains are increasing.^96^^,^^165^^,^^169^ The key issue is the sensitivity of the detection stage, since silicon detectors—e.g., common CCD or CMOS cameras—become almost blind above 1000 nm, and longer wavelength detectors—e.g., indium-gallium-arsenide—are more costly, noisy, and often require cooling. Nevertheless, investigation of the feasibility if an estimation method has enough sensitivity for the tissue optical properties at longer wavelengths should be carried out before implementation.

The final limiting factor is the speed. Slow measurement and processing speed hinders real-time applications, which are of high importance in clinical settings, such as surgical guidance where clinicians want to have real-time indications of tissue optical properties. As discussed in Secs. 4.1–4.5, efforts have been made to improve the measurement speed by the use of faster electronics and algorithms. In addition, high processing speed is desirable to generate measurement results instantly. Analytical closed-form solutions giving $\mu_{a}$ and $\mu_{s}^{\prime }$ based on the diffusion theory are not always available and have limited accuracy due to the diffusion approximation. Finding $\mu_{a}$ and $\mu_{s}^{\prime }$ by iteratively computing the forward model is possible with the AD method as implemented in the IAD algorithm but is impractical for the MC simulation given its low computational speed. As a result, it is more often to find $\mu_{a}$ and $\mu_{s}^{\prime }$ by interpolating the precomputed lookup table (see Secs. 4.1–4.5). Recently, seeking a faster mapping from the measured signal to $\mu_{a}$ and $\mu_{s}^{\prime }$, machine learning and deep learning have started being explored.^132^^,^^196^^–^^199^

## 6 Summary and Outlook

This tutorial focuses on two dominant tissue optical properties, namely, absorption and scattering coefficients, and the estimation methods that extract these properties using inversion models of light–tissue interactions. We presented the estimation methods according to their measurement domains and compared them in terms of measurement capability, inversion method, accuracy, acquisition speed, system size, and cost, in order to help the reader choose the estimation technique that best suits their needs. Measurement principles and practical considerations are discussed, with links to how-to guides provided, for readers who are interested in implementing the measurement systems. In the future, with access to low-cost small-footprint systems and standard phantoms, as well as the emergence of commercial measurement products, the applications of tissue optical property estimation are highly likely to expand even more widely, having greater impacts on clinical healthcare and homecare. More accurate, precise, and faster measurements call for improved standardization of calibration phantoms, investigation of methods for avoiding tissue contact and reducing the dependence on sample homogeneity assumption, as well as the development of faster electronics, algorithms, and computations. Moreover, measurements that can estimate additional tissue chromophores will bring more opportunities to investigate new biomarkers.

## Data Availability

This tutorial does not contain any data that is not publicly available. The data to replicate Figure 1 can be accessed using the references provided in the figure caption.
